# Photosynthetic variation and responsiveness to CO_2_ in a widespread riparian tree

**DOI:** 10.1371/journal.pone.0189635

**Published:** 2018-01-02

**Authors:** Shannon Dillon, Audrey Quentin, Milos Ivković, Robert T. Furbank, Elizabeth Pinkard

**Affiliations:** 1 Genetic Diversity and Adaptation, Breakthrough genetic technologies for crop productivity, CSIRO Agriculture and Food, Canberra, ACT, Australia; 2 Landscape Intensification, CSIRO Land and Water, Hobart, TAS, Australia; 3 ARC Centre of Excellence for Translational Photosynthesis, Research School of Biology, Australian National University, Acton, ACT, Australia; Wageningen University, NETHERLANDS

## Abstract

Phenotypic responses to rising CO_2_ will have consequences for the productivity and management of the world’s forests. This has been demonstrated through extensive free air and controlled environment CO_2_ enrichment studies. However intraspecific variation in plasticity remains poorly characterised in trees, with the capacity to produce unexpected trends in response to CO_2_ across a species distribution. Here we examined variation in photosynthesis traits across 43 provenances of a widespread, genetically diverse eucalypt, *E*. *camaldulensis*, under ambient and elevated CO_2_ conditions. Genetic variation suggestive of local adaptation was identified for some traits under ambient conditions. Evidence of genotype by CO_2_ interaction in responsiveness was limited, however support was identified for quantum yield (φ). In this case local adaptation was invoked to explain trends in provenance variation in response. The results suggest potential for genetic variation to influence a limited set of photosynthetic responses to rising CO_2_ in seedlings of *E*. *camaldulensis*, however further assessment in mature stage plants in linkage with growth and fitness traits is needed to understand whether trends in φ could have broader implications for productivity of red gum forests.

## Introduction

Forest trees are foundation species in ecosystems worldwide. They are long lived, often wide spread and traverse strong environmental gradients. As a result, forest tree species frequently exhibit adaptive phenotypic clines reflecting genetic adaptations to local environment [1,2]. Such clines highlight the capacity of forests to adapt to their environment over evolutionary time scales [3], however it is less is well understood how forests will adapt to future climate change [4]. Increasing concentration of atmospheric carbon dioxide (CO_2_) is one of the most important global change pressures currently affecting forests, which acts directly through its effect on leaf-level gas exchange, and indirectly through its effect on climate [5]. How forest species respond to shifts in rising CO_2_, in interaction with broader climate variation, will have consequences for the ecological communities which they support, as well as restoration and commercial forestry [6,7].

As more species confront environmental change, it is becoming important to quantify the factors influencing their capacity to adapt and to monitor these [8,9,10]. Adaptive responses to CO_2_ in trees could include both evolutionary adaptations and phenotypic plasticity, and a better understanding of these effects will assist management of future forests [11,12]. The ability of an organism to change its phenotype in response to changing environment, or plasticity, is a widely recognised adaptive mechanism in plants [13,14,15], that could have particular utility mediating phenotypic adaptation in forest tree species with long generation times where rates of evolutionary adaptation may be slow compared to the velocity of environmental change [4,16]. Plastic responses will therefore be highly relevant to adaptation in forests trees over the time frame in which CO_2_ is projected to increase [17].

Plastic responses of morphological and physiological traits under CO_2_ enrichment are extensively documented in forest trees, including eucalypts [18,19,20,21,22,23,24,25]. This generally points to increased productivity and improved water use efficiency of forests driven by CO_2_ fertilisation [5,26,27,28]. However the extent to which CO_2_ stimulation effects vary among genotypes or populations, and subsequent impacts for forest productivity across a species range, is not well understood. Genetic effects determining CO_2_ response, or genotype by CO_2_ interaction (G × CO_2_), have been quantified in other plants [7,29,30,31,32,33], suggesting that populations or genotypes can respond in ways not predicted from generalised interpretations of CO_2_ response.

Characterisation of G × CO_2_ responses in trees is therefore warranted, and may be furthered by better understanding the processes leading to genetic variation in response. Evolutionary adaptation has been proposed as one constraint on plasticity (or adaptive plasticity) in plants where trends in phenotypic response reflect adaptation along environmental clines in nature [12,34,35,36]. Due to the breadth of environments encountered by widely distributed forest tree species *in situ*, local adaptation is expected to be a driver of variation in plasticity, and this has been observed for phenology, leaf and physiological traits [37,38,39]. It is less well understood whether pre-existing adaptations to environment underlie variation in population plasticity in physiological responses to CO_2_, although this has been suggested [7,40].

To address gaps in our understanding of population level adaptation to CO_2_ in forest trees, we investigated the extent of local adaptation and responsiveness to elevated CO_2_ for key photosynthesis traits in a wide spread, ecologically and genetically diverse eucalypt, *E*. *camaldulensis*. Traits were assessed across the species natural distribution, where we firstly explored whether genetic variation among provenances and subspecies was detectable and if so whether this variation was likely to have been influenced by adaptation to local environment. We subsequently investigated the degree to which variation in photosynthetic responses to CO_2_ enrichment was dependant on provenance of origin, and where G×CO_2_ was identified, whether there was evidence that trends in plasticity could have been constrained by pre-existing adaptations to local environment.

## Methods and materials

### Genetic material

**Fig 1:**
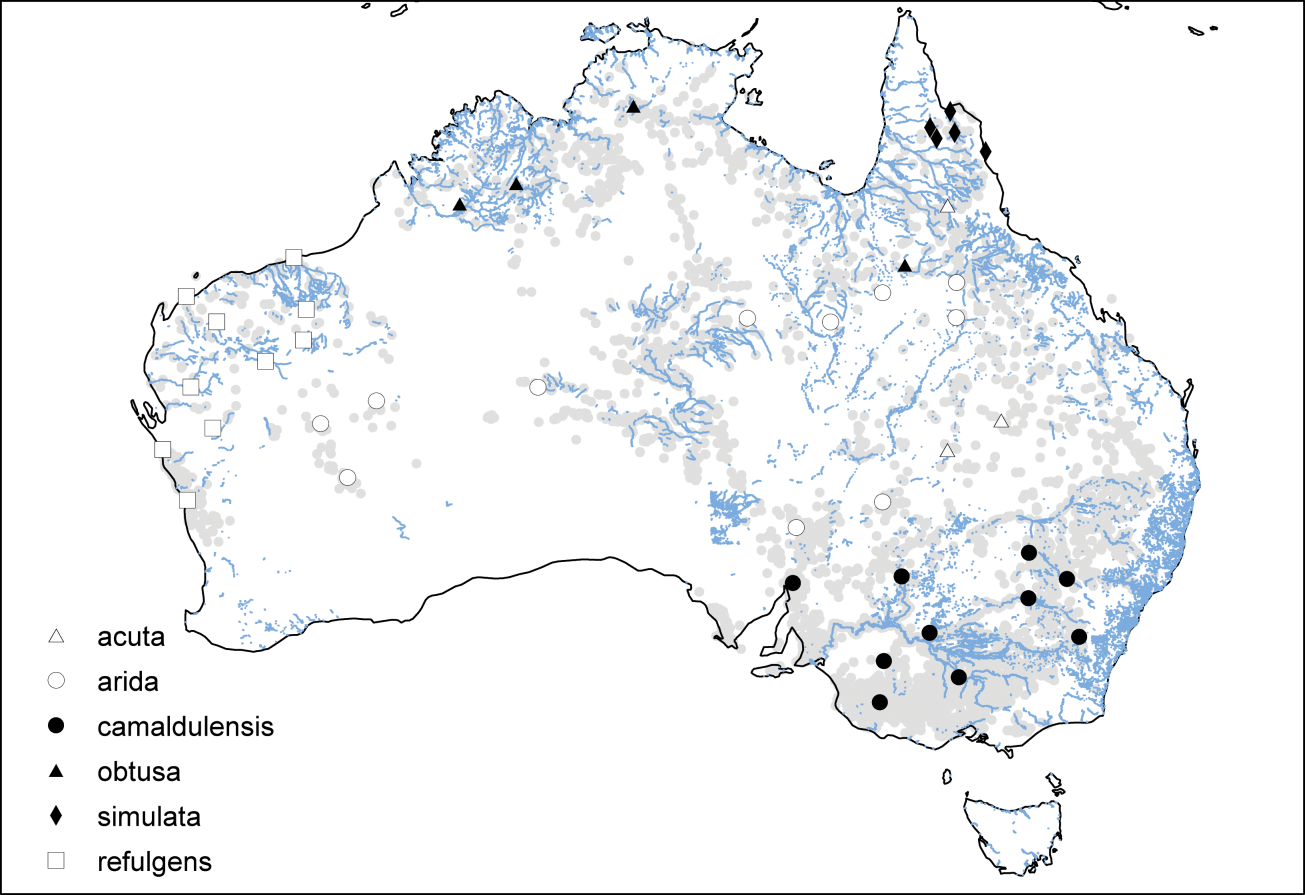
Distribution of *E*. *camaldulensis* provenances sampled in this study. Occurrence records (underlaid) spanning the species natural range were obtained from the Atlas of Living Australia (http://www.ala.org.au. Accessed 12 May 2016). National Surface Hydrology Polygon obtained from Geoscience Australia [82]. Figure produced using ArcGIS v. 10.3.

In total 486 *E*. *camaldulensis* genotypes representing 43 provenances and 5 subspecies were sampled with between 5 and 12 genotypes per provenance for 401 “test” cases, and an additional 85 “control” plants with an average of 2 plants per provenance (Fig 1, Table 1). Genotypes were sampled across the natural range of *E*. *camaldulensis*, in an attempt to capture a representative sample of genetic diversity for this species. *E*. *camaldulensis* seed was obtained from the Australian Tree Seed Centre (Canberra, Australia) as provenance seed lots collected from individual mother trees *in situ*, with the exception of four seed lots for which seed was bulked. Within provenance each genotype represented a different seed lot, thus the design did not capture within family variation. Seeds were germinated in a native low phosphorus potting mix at ambient CO_2_ (400 ppm; 28°C) on a 16hr day/night light cycle. Four weeks post germination seedlings were transferred into 10cm diameter 0.75L planter bags with a native low phosphorus potting mix (1/3 of river sand, 1/3 of peat moss and 1/3 of natural compost) and moved to a glasshouse under similar atmospheric conditions (400 ppm; 24°C) and a natural 12hr day/night light cycle. Two applications of a systemic fungicide (Fongarid®, active constituent: 250g/kg Furalaxyl; dilution: 1g/L) were applied at 2 week intervals from approximately 5 weeks until 2 months of age to eliminate risk of root fungal disease.

**Table 1:** Provenances and subspecies of *E*. *camaldulensis* sampled for assessment at ambient and elevated CO_2_.

provenance name	sub species	state	longitude	latitude	no. test individuals	no. control
EINASLEIGH RIVER	*acuta*	QLD	144.01	-18.11	5	1
WARDS RIVER	*acuta*	QLD	146.06	-26.29	7	1
BULLOO RIVER	*acuta*	QLD	144	-27.42	4	0
GILES CREEK	*arida*	WA	128.4	-25.03	9	1
MELROSE	*camaldulensis*	SA	138.12	-32.48	9	1
CONDOBOLIN	*camaldulensis*	NSW	147.09	-33.06	11	1
MENINDEE	*camaldulensis*	NSW	142.26	-32.23	9	1
LAKE ALBACUTYA	*camaldulensis*	VIC	141.58	-35.45	10	2
ORD RIVER	*obtusa*	WA	127.58	-17.28	11	2
N. FITZROY CROSSING	*obtusa*	WA	125.42	-18.06	10	1
PETFORD AREA	*obtusa*	QLD	-17.24	145.02	0	2
DE GREY RIVER	*refulgens*	WA	119.11	-20.1	11	2
NEWMAN	*refulgens*	WA	119.47	-23.24	9	1
MEEBERRIE	*refulgens*	WA	116.03	-26.59	12	2
LAURA RIVER	*simulata*	QLD	144.28	-15.34	11	2
ARTHUR CREEK	*arida*	NT	136.38	-22.41	12	2
BOULIA	*arida*	QLD	139.55	-22.55	10	2
GLEN GORGE CREEK	*arida*	QLD	141.53	-21.44	8	1
MUTTABURRA	*arida*	QLD	144.33	-22.38	12	2
BAROOTA WATERHOLE	*arida*	QLD	144.35	-21.05	10	1
EMU CREEK	*arida*	SA	138.24	-30.38	10	2
TIBOOBURRA	*arida*	NSW	141.53	-29.4	10	2
PALMER RIVER N	*simulata*	QLD	143.6	-15.56	11	2
LAKEFIELD NP	*simulata*	QLD	144.11	-14.53	10	1
PALMER RIVER S	*simulata*	QLD	145.46	-16.06	13	2
MOREHEAD RIVER	*simulata*	QLD	143.34	-15.15	10	2
BIDGEMIA	*refulgens*	WA	115.19	-25.02	10	2
GORGE JUNCTION	*refulgens*	WA	118.03	-24.04	11	2
KOOLINE	*refulgens*	WA	116.17	-22.54	8	1
MINDEROO	*refulgens*	WA	115.01	-21.57	9	1
NULLAGINE CREEK	*refulgens*	WA	119.58	-22.07	5	1
KATHERINE RIVER	*obtusa*	NT	132.04	-14.33	10	2
LENNARD RIVER	*obtusa*	WA	-16.3	124.3	0	4
WYNDHAM	*obtusa*	WA	-15.31	128.12	0	6
VICTORIA RIVER	*obtusa*	NT	-16.2	131.07	0	5
N. OF MAXWELTON	*obtusa*	QLD	142.38	-20.38	5	1
WELLINGTON	*camaldulensis*	NSW	148.56	-32.33	6	1
EUCHCA MURRAY RIVER	*camaldulensis*	VIC	144.44	-36.07	9	1
BALRANALD	*camaldulensis*	NSW	143.33	-34.38	10	1
NARRANDERA	*camaldulensis*	NSW	-34.45	146.33	0	6
YASS RIVER	*camaldulensis*	NSW	149.02	-34.53	10	1
NYNGAN	*camaldulensis*	NSW	147.11	-31.33	8	1
DOUGLAS	*camaldulensis*	VIC	141.43	-37.02	10	1
WIMMERA R-ELMHURST	*camaldulensis*	VIC	-37.13	143.16	0	2
MURCHISON RIVER	*refulgens*	WA	114.11	-27.4	10	2
ARROWSMITH LAKE	*refulgens*	WA	115.05	-29.33	10	2
STATION CREEK	*arida*	WA	121.15	-28.47	7	1
LAKE WAY	*arida*	WA	120.12	-26.42	9	1
SW CARNEGIE	*arida*	WA	122.25	-25.55	10	1

### Growth conditions

At approximately 2 months of age 486 seedlings were transferred to four controlled-environment growth chambers (PGC20 Conviron®, Winnipeg, Canada) using a randomised design. Plants were arranged in 350 x 300 mm plastic seedling trays, at nine plants per tray. Every second week over the course of the experiment, trays within each cabinet were rotated to reduce confounding position effects rather than blocking. At this time Aquasol® soluble plant fertiliser (NPK- 23:3:95 and 14 trace elements) was provided at half strength, and subsequently every month for the duration of the experiment to ensure plants were not nutrient limited. Temperature regime was set to 18°C (night) and 24°C (day), which enveloped at least one of annual mean, maximum or minimum temperature encountered naturally by any of the sampled provenances. A 16hr day/night photoperiod was applied, to approximate the natural photoperiod encountered between 37–14°S and 115–149°E. Each cabinet was fitted with Growlux fluorescent lamps (Sylvania 24W/T5/GRO, Australia) and a black-light (Sylvania FHE 28W/T5/BLB, Australia) to provide light in the red and blue regions of the spectra coinciding with the photosynthesis action spectrum, while enriching with high frequency light in the UVa spectrum recommended for normal growth of eucalypts [41]. Light intensity was implemented via an hourly step function in the morning and evening to simulate natural light conditions. Plants experienced full light, corresponding to about 1000 μmol m^-2^ s^-1^, for 7 hours each day. Each cabinet was submitted to the same photoperiod but was delayed by 2 hours between chambers to allow an intensive measurement campaign. Relative humidity in the cabinet was controlled at 50% during the day and 60% during the night by a dehumidifier (Belta 601). Plants were watered to saturation from the base daily for the first six weeks, and twice daily from seven weeks.

### CO_2_ conditions

The cabinet experiment aimed to detect photosynthetic variation among provenances under ambient CO_2_ conditions, and to assess evidence of provenance by environment interactions (G × E) in response to elevated CO_2_ conditions. Plants were sequentially exposed to ambient [CO_2_] (aCO_2_, 400 ppm) over 10 weeks followed by elevated [CO_2_] (eCO_2_, 800 ppm) for a further 8 weeks (± 20 μmol CO_2_ mol^−1^). This included a period of two weeks to allow plants to acclimatise to the cabinets before commencing the ambient treatment. The chosen CO_2_ levels for each treatment were based on current and projected (yr. 2100) atmospheric CO_2_ levels respectively (IPCC 2014). To maintain CO_2_ at the desired concentration, a non-dispersive CO_2_ analyser (GMT220 Vaisala Carbocap® CO_2_; Vantaa; Finland) continuously measured and directly controlled CO_2_ in each chamber via injection of industrial grade compressed CO_2_. This was combined with a granular soda lime-based CO_2_ controller. In total 401”test” genotypes were subjected to both aCO_2_ and eCO_2_ phases. The sequential design of the cabinet treatments accounted for genotype effects between CO_2_ treatments. To account for potential ontological effects due to the treatments being measured eight weeks apart, a set of 85 “control” individuals (Table 1) were retained at aCO_2_ for the duration of the experiment.

### Phenotypic data

Previous studies in trees have established that photosynthetic traits are responsive to changing CO_2_, and could therefore serve as a suitable base for assessing intraspecific variation in CO_2_ response. Variation in photosynthetic processes also has potential to impact growth, productivity and fitness of individual trees or forest stands, and therefore are an important trait in the context of forests growing under future CO_2_ conditions. A set of ten photosynthesis traits were estimated on test and control plants during the final week of each treatment phase, or within the final two weeks in the case of integrated photosynthetic traits. All measurements were performed on a fully expanded, mature leaf from the upper crown of test and control plants that had developed during the respective treatment phase. By standardising sampling of leaves at a common developmental stage we attempted to limit confounding ontological variation between treatment time points. Gas exchange measurements were performed using a Li-Cor LI-6400 portable photosynthesis instrument (Li-Cor, Inc., Lincoln, USA) within the period of peak photosynthetic activity, determined from diurnal measurements (0900–1200 hrs). Leaves in the cuvette were illuminated to saturating photon flux density (PFD) of 2000 μmol m^-2^ s^-1^, and ambient temperature (24°C). Leaves were measured at an external CO_2_ concentration of 400 ppm (growth CO_2_) during aCO_2_ treatment, and sequentially at both 400 ppm and 800 ppm (growth CO_2_ for control and test plants respectively) during the eCO_2_ treatment. Measuring gas exchange at two levels in the test and control plants in the elevated treatment enabled us to examine the effect of CO_2_ acclimation vs instantaneous enhancement of photosynthesis. In each instance light-saturated assimilation rate (*A*_net_, μmol m^−2^ s^−1^) was recorded after an equilibration period of up to five minutes.

The response of net assimilation rate (*A*) to varying intercellular CO_2_ concentrations (*A*–*C*_i_), and varying light intensities (*A*-light) were also determined in the seventh week of both CO_2_ treatments. Integrated measurements of photosynthesis are expected to be less variable (greater precision) than instantaneous measures (such as A_net_), and can provide insight into biochemical processes underpinning CO_2_ assimilation. However due to the time required for these measurements this was performed on a subset of plants, 3–4 per provenance, and did not include control plants. In the ambient treatment this totalled 131 plants and in the elevated 124 plants. The *A-C*_*i*_ curve consisted of 15 steps of external CO_2_ concentrations applied in succession over 400, 350, 250, 150, 100, 50, 0, 400, 500, 750, 900, 1200, 1500, 2000, 400 ppm. Leaf photosynthesis was then measured at 12 photon flux densities over 2000, 1800, 1500, 1000, 800, 600, 400, 200, 100, 75, 50, 0 μmol m^−2^ s^−1^. Dark respiration was defined as the absolute CO_2_ exchange rate measured during the last step of the *A*-light curve. Leaves were allowed to equilibrate for 5 minutes between each step of the *A-C*_*i*_ and *A-*light curves. All measurements were performed at ambient temperature (24°C) with VPD held close to 1 kPa. Biochemical parameters were calculated from the *A-C*_*i*_ (V_cmax_, J, TPU, Γ) and *A-*light (A_max_, φ, R_dark_, LCP, θ) curves for each genotype using established photosynthesis model equations [42](S1 Table). Because of the potential for leaf temperature to influence estimates of quantum yield [43], the relationship between leaf temperature (T_leaf_) and φ at time of measurement was assessed but found not to be correlated at either ambient or elevated CO_2_ (aCO_2_: R^2^ = 0.002, p = 0.56; eCO_2_: R^2^ = 0.01, p = 0.249). As a quality measure *A-C*_*i*_ and *A-light* curves are presented for a subset of plants at both CO_2_ levels in S1 Fig. Instantaneous light-saturated assimilation rate (A_net_) was also taken from the *A-C*_*i*_ curve at 400ppm in the aCO_2_ and eCO_2_ treatments to facilitate direct comparison with integrated biochemical traits. Trait data for all genotypes across the two CO_2_ treatments and controls have been made available in supplemental S1 File.

### Statistical methods

#### Photosynthetic variation among provenances and subspecies

Variation in photosynthetic traits among provenances and subspecies at ambient CO_2_ was first assessed via linear discriminant analyses of principal components, by applying subspecies as the grouping factor, to identify a set of multivariate discriminant functions that maximise variance among provenances, using the MASS package in R [44]. Principal components applied in this analysis were first generated on raw trait data across individuals using the “prcomp” function in base R (R Development Core Team, 2015) (S2 Table). Significance of subspecies variance across discriminant functions was assessed via MANOVA with a Wilks' Lambda test in base R. Discriminant functions and their coefficients were visualised using the ggord package in R [45].

Quantitative variation in photosynthetic traits among provenances and subspecies at ambient CO_2_ was subsequently examined to assess the contribution of genetic factors to trait variation across the species range. Trait data was assessed for incorrect entries and outlying values. To estimate the proportion of phenotypic variance at ambient CO_2_ attributable to genetic groups, or provenance effect, random effect variance components were estimated in a bivariate linear mixed model analysis as per Falconer and McKay (1996), implemented in ASReml-R Release 3 (Butler *et al*. 2009, R Development Core Team, 2015):

y=Xb+Zu+e

where ***y*** is the vector of trait observations, ***b*** and ***u*** are vectors of fixed (cabinet and CO_2_) and random (provenance) effect estimates respectively, ***X*** and ***Z*** are incidence matrices for fixed and random model terms and ***e*** is a vector of random residual effects. The proportion of the total phenotypic variance (or provenance effect) (*P*_*mr*_) explained by the random provenance effect variance components ($\sigma_{p}^{2}$) at ambient CO_2_, was subsequently estimated following Falconer and Mackay (1996):

$$P_{mr}=\frac{\sigma_{p}^{2}}{\sigma_{p}^{2}+\frac{\sigma_{e}^{2}}{n}}$$

where $\sigma_{e}^{2}$ is the residual error variance component at ambient CO_2_ and n is the harmonic mean of the number of seedlings per provenance. The latter was applied to account for unbalanced data. Similar analyses were performed at the subspecies level. Provenance least squares means (or best linear unbiased estimates–BLUEs) were estimated for each trait under ambient conditions by fitting provenance as a fixed term in the linear model framework already described, and applying the predict() function in ASReml-R. This provided an adjusted mean (BLUE) for each provenance accounting for potential sources of variation in the data, subsequently applied to relate phenotypic values to environment at site of origin (see next section), deemed necessary since environmental estimates were aggregated at the provenance level. Applying the same linear model framework described above, without random effects, associations between provenance BLUEs among traits were fitted using the “lm” function in base R.

#### Environmental associations

Associations between traits and environmental variables were explored, to assess potential for local adaptation to explain patterns of phenotypic variation among populations. Point estimates for environmental variables including climate, ecology and geological variables were obtained for each provenance based on geographic coordinates from the Atlas of Living Australia [46]. Principal component analyses was performed on this data set to reduce dimensionality, grouping parameters by: climate (37 variables: including rainfall, evaporation, temperature, humidity, wind and irradiance), geology (11 variables: including nutrient availability, slope, soil depth, erosion, and weathering) and ecology (4 variables: NPP, NDVI, endemism and species richness), using the “prcomp” function in base R. This produced a set of 6 orthogonal “environmental” axes explaining up to 50% of the cumulative variance (S3 Table). Associations with environment were performed using provenance BLUEs for each trait. Collinearity was detected among traits at ambient CO_2_, therefore we also performed environmental associations with a set of uncorrelated phenotypic variables produced via a principal components analysis upon provenance trait BLUEs using the “prcomp” function in base R. This produced a set of 3 orthogonal “phenotypic” axes, with an eigen value > 1 explaining at least 50% of the cumulative variance (S4 Table). Associations between traits or PC variables and environmental point estimates were fitted in a linear model framework using the lm() function in base R. To account for potentially neutral demographic effects on phenotypic variation among provenances the analysis was performed with and without geographic coordinates (latitude and longitude) as an additional fixed term, with the caveat that this will only account for isolation by distance.

#### Responses to CO_2_

Analyses of overall trait responsiveness to CO_2_ treatment was performed separately for test and control plants, implemented in a simple linear model framework using the lm() function in base R, where treatment (ambient or elevated) and cabinet were included as a fixed terms in the model. Significance of treatment effect was obtained for each trait using the anova() function and treatment least squares means were estimated from the model intercept with the R package lsmeans [47].

Provenance by CO_2_ interaction (G × E) was examined in test plants via cross treatment genetic correlation (gCor), where restricted maximum likelihood variance (REML) and correlations for random provenance effects across CO_2_ treatments were derived using ASReml-R Release 3, using the linear mixed model framework previously described. This was implemented by fitting random effects across CO_2_ treatments in an unstructured correlation matrix, assuming heterogeneous variance estimates, using the corgh() function. In this way the provenance level genetic correlation was calculated for the same trait in two different CO_2_ environments. All effects were assumed to have heterogeneous variances across treatments, and variance correlations were estimated both for provenance and for error terms. REML derived variances and correlations were constrained to fall within the theoretically possible range from -1 to +1. Non-parametric correlation of provenance least-square BLUEs between the ambient and elevated treatments were also assessed via a Spearman’s Rank Correlation test. In the elevated treatment provenance BLUEs were estimated in same way as described for the ambient treatment. The trait reaction norm, or Δtrait, was estimated as a measure of plasticity for each provenance from the ratio of elevated to ambient treatment provenance least-square BLUEs, where negative values indicate a decrease in trait estimates in the elevated treatment. To assess possible mechanistic drivers of variation in plasticity where G × E interaction was detected, provenance level associations between Δtrait and environmental point estimates were fitted in a linear model framework using the lm() function in base R, with and without geographic coordinates (latitude and longitude) as an additional fixed term to account for potentially neutral demographic effects.

## Results

### Photosynthetic variation at ambient CO_2_

**Fig 2:**
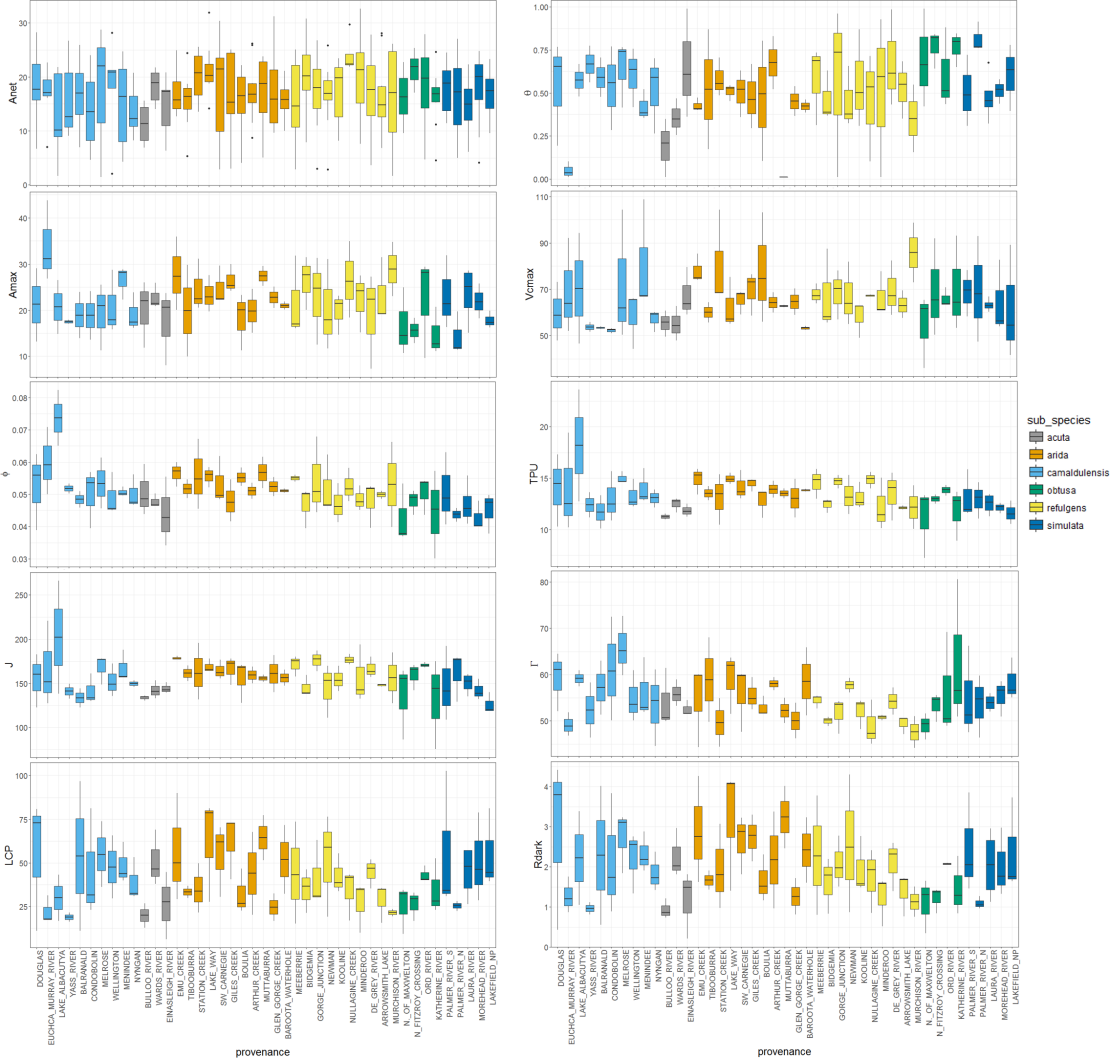
Box plots illustrate variation among provenances, grouped by subspecies, for each photosynthetic trait, presented as the mean, 1^st^ and 3^rd^ quartiles of the distribution and outliers within whiskers spanning 1.5 times the interquartile range (IQR). Subspecies are ordered based on their approximate south to north latitudinal position.

At ambient CO_2_ significant provenance and subspecies variation was detected, pointing to genetic factors contributing to variation in some traits across the species range. Linear discriminant analyses (LDA) identified a weak cline based on a single significant discriminant function maximising variation in photosynthetic traits at the subspecies level (S2 Fig; Wilks test p = 0.009). The discriminant function coefficients (PC1 = 0.565, PC2 = -0.009 and PC3 = -0.132) identified PC1 as primarily contributing to variation among subspecies and implicated a set of traits loading to PC1 with a correlation of 0.5 or greater (S2 Table), including φ, J, TPU, LCP, V_cmax_, A_max_ and R_dark_. In the mixed model analysis a significant proportion of phenotypic variation was explained by subspecies for several photosynthetic traits, ranging from 44 to 65 percent, namely φ, J, LCP, Γ and R_dark_ (Table 2), most of which were previously implicated in the LDA. In general, aggregating variance estimates at the subspecies level improved estimation of genetic effects for photosynthetic traits, with significant variation detected at the provenance level for a single trait only, J, suggesting insufficient replication to adequately capture provenance level effects for most traits. In the case of φ, θ, Γ and R_dark_ a moderate proportion of trait variation (13 to 22%) was explained by provenance, however large standard errors relative to these estimates deemed them insignificant. In other cases variance at the provenance level was detected relative to the standard error, but variance explained was too small to be of practical significance. Variance of raw phenotypic values within provenances grouped by subspecies for all ten traits is additionally presented (Fig 2). Covariation of provenance means under ambient CO_2_ identified relationships between biochemical parameters, which reflect known mechanistic dependencies (S5 Table, S3 Fig). For example, variation in quantum yield (φ) was strongly positively correlated with the maximum rate of CO_2_ fixation (A_max_), instantaneous CO_2_ assimilation (Anet), rate of electron transport (J) and maximum rate of carboxylation (V_cmax_).

**Table 2:** Phenotypic variance, provenance and subspecies effects, for 10 photosynthetic traits under ambient CO_2_.

	provenance			subspecies		
trait	*σ*_*p*_^*2*^	*σ*_*e*_^*2*^	*P*_*mr*_	*σ*_*p*_^*2*^	*σ*_*e*_^*2*^	*S*_*mr*_
Anet	1.97e-07	4.07e+01	4.02e^-08^±2.90e^-09^	0.13	40.54	0.10±0.25
A_max_	8.48e-07	4.50e+01	6.17e^-08^±8.03e^-09^	1.67e+00	4.37e+01	0.34±0.36
φ	4.34e-06	5.79e-05	0.20±0.26	7.70e-06	5.54e-05	0.65±0.20*
J	47.58	542.29	0.22±0.17*	38.73	557.61	0.48±0.29*
LCP	4.98	443.38	0.04±0.30	26.64	429.58	0.45±0.32*
θ	4.23e-03	5.05e-02	0.21±0.25	1.43e-03	5.35e-02	0.26±0.41
V_cmax_	1.33e-06	1.88e+02	2.31e^-08^±3.00e^-09^	1.88e-06	1.88e+02	1.30e^-7^±1.70e^-8^
TPU	2.44e-07	4.09e+00	1.91e^-07^±2.48e^-08^	0.51	3.71	0.64±0.21*
Γ	2.99	38.65	0.20±0.21	2.34	39.67	0.44±0.33*
R_dark_	0.04	0.97	0.13±0.22	8.40e-02	9.43e-01	0.54±0.26*

*σ*_*p*_^*2*^ *= random effect phenotypic variance; σ*_*e*_^*2*^ *= residual variance*. *P*_*mr*_ and *S*_*mr*_ = effect of random provenance or sub-species means under ambient conditions ± standard error

* = proportion of variance attributable to provenance or subspecies significant within ± one standard error

**Fig 3:**
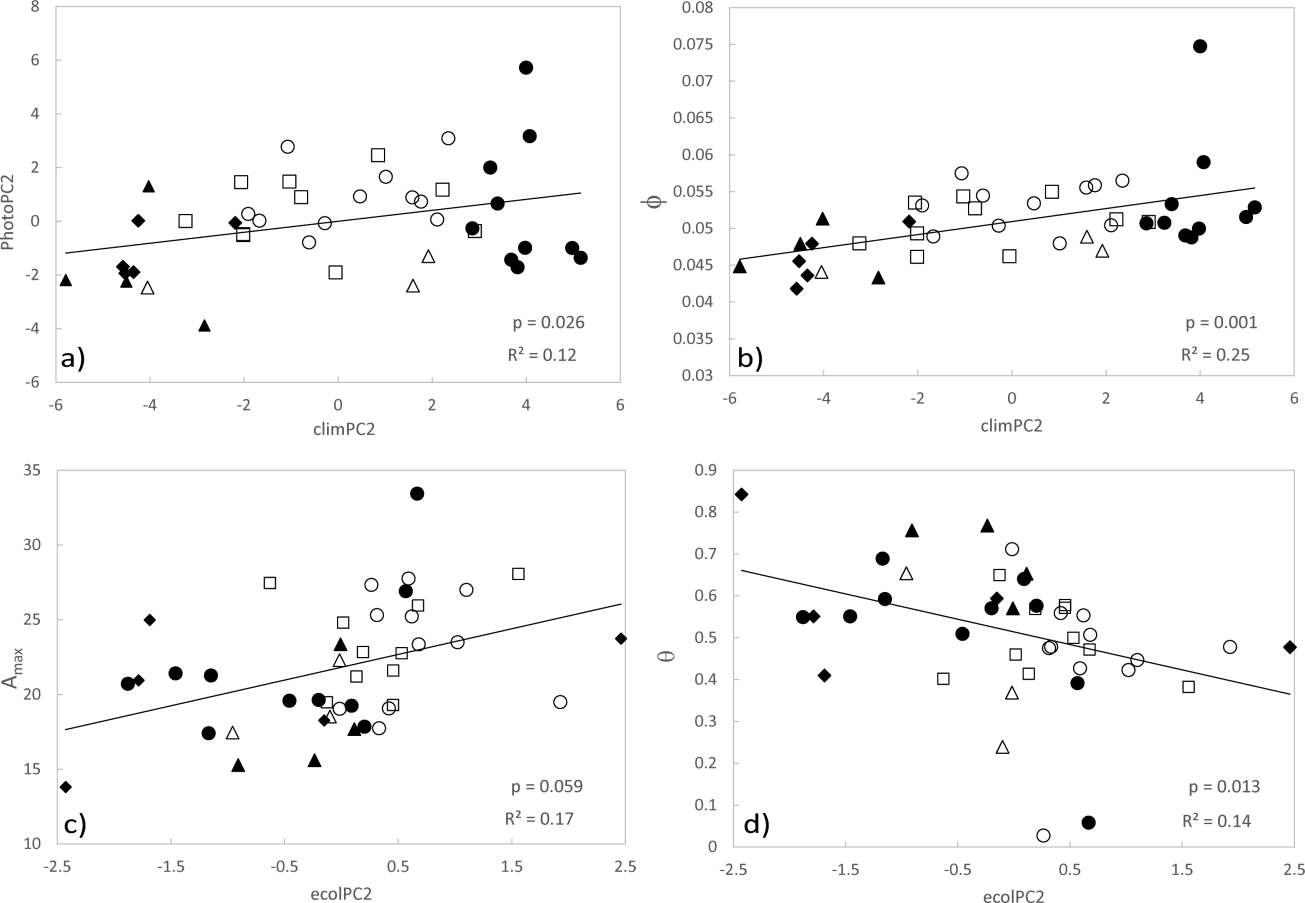
Association of provenance BLUEs (least square means) for selected traits and climate parameters. For principal components, arrows against the y axis indicate the relative shift in environmental variables based on loadings with increasing values of the PC estimate. Likewise arrows against the vertical axis indicate relative shift in trait values based on loadings with decreasing values of the PC estimate.

Genetic variation in photosynthetic traits detected among provenances and subspecies under current CO_2_ conditions point to underlying genetic differences in photosynthesis that may have been driven by adaptation to environment, such as climate or geological factors, given red gum is a widespread and environmentally heterogeneous species. Local adaptation was subsequently invoked as one possible explanation for the observed phenotypic variation in several traits based on associations with environmental parameters (Table 3, Fig 3). The results suggest a relationship between environmental parameters loading to climPC2 (precipitation, temperature, seasonality, water stress index and wind; S3 Table) and traits loading to traitPC2 (φ, A_max_, J and TPU; S4 Table), where increasing values of climPC2 correspond with higher winter rainfall, lower temperatures, and increasing values of traitPC2 correspond with increasing φ, J, A_max_ and TPU (Fig 2A). With the exception of A_max_, an adaptive model for these traits is consistent with the genetic component implied from significant subspecies effects. Associations with climPC2 were also observed for individual traits loading to photoPC2 (Fig 2B, S4 Table). In addition ecological variables loading to ecolPC2 were associated with A_max_, V_cmax_ and θ(Fig 2C and 2D). Here maximum assimilation rate was correlated with higher endemism and decreased NDVI, with the inverse relationship detected for curvature of the light-response curve. In several cases associations with climate and ecology factors were diminished after accounting for spatial factors, possibly indicating demography rather than adaptation as influencing the observed patterns, however local adaptation cannot be ruled out as a driver because environmental parameters loading to climPC1, climPC2 and ecolPC2 are significantly spatially autocorrelated (climPC1, R^2^ = 0.57, p < 0.001; climPC2, R^2^ = 0.87, p < 0.001; ecolPC2, R^2^ = 0.26, p < 0.001).

**Table 3:** Association of provenance level trait variation (BLUEs), including multivariate PC’s, with environmental parameters under ambient CO_2_.

trait	environment	slope	R^2^	F	p	F^†^	p^†^
J	climPC1	-1.14	0.10	4.5	0.039*	0.96	0.332
photoPC2	climPC2	0.20	0.12	5.4	0.026*	3.44	0.071·
Amax	climPC2	0.38	0.08	3.8	0.059·	1.14	0.292
φ	climPC2	1.59e-7	0.25	13.9	0.001***	11.55	0.002**
TPU	climPC2	0.13	0.10	4.7	0.037*	4.30	0.044*
Amax	ecolPC2	1.72	0.17	8.5	0.006**	1.17	0.286
θ	ecolPC2	-0.06	0.14	6.8	0.013*	2.74	0.106
V_cmax_	ecolPC2	2.32	0.09	4.0	0.052·	1.77	0.191

Associations based on a single level fixed effect linear model. R^2^ = square of Pearson’s R for the model. F = ANOVA F-statistic; p = probability of model

† model controlling for geographic coordinates

* = significant at p < 0.05

** significant at p < 0.01

*** significant at p < 0.001

**·** = marginally significant

### Photosynthetic responses to CO_2_

Overall response between CO_2_ treatments for each trait is presented in Table 4. Significant downregulation of net photosynthesis (A_net_^[400–400]^) in test plants at elevated growth conditions relative to ambient conditions was detected, with no change observed in the corresponding controls measured at the same external [CO_2_] in the cuvette. Conversely, instantaneous enhancement in net photosynthesis (A_net_^[400–800]^) was observed in both test and control plants at the second treatment point relative to the ambient treatment when measured at 800ppm [CO_2_] in the cuvette. A lack of change in A_net_ between treatments for control plants indicated that ontological effects between treatment points were not likely to confound interpretation of photosynthetic responses to CO_2_, in line with the expectation that age related shifts in photosynthesis traits of woody perennials are limited to major developmental transitions [48]. Although this comparison could not be made at the provenance level, because of insufficient replication of control plants within provenances, variation in response ratio (elevated A_net_^[400]^/ambient A_net_^[400]^) for individual plants indicated that overall response is not likely to mask genotypic variation in net photosynthesis response to elevated CO_2_. This was shown by way of a simple one sample t-test, which for control plants indicated the distribution of response ratio across samples was not significantly different to 1 (theoretical mean for a distribution based on plants with no CO_2_ response) at p < 0.05, whereas deviation from this limit among test plants was highly significant (p = 4.72e^-15^) (S4 Fig). Shifts in *A-C*_*i*_ and *A-*light curve derived photosynthetic traits were nominally treated as CO_2_ effects on the basis that these parameters will relate to changes in A_net_. This is supported by downregulation of all biochemical traits in the elevated treatment relative to ambient, although this shift was not significant in the case of Γ (Table 4). Estimates of overall A_net_ taken from the *A-C*_*i*_ curve at 400ppm in both the ambient and elevated treatments also indicated a significant downturn in photosynthetic activity under CO_2_ enrichment (aCO_2_^μ^ = 18.15, eCO_2_^μ^ = 16.26, F = 8.34, p = 0.004).

**Table 4:** Trait response to CO_2_ across ambient (aCO_2_) and elevated (eCO_2_) treatments.

trait	aCO_2_^μ^	eCO_2_^μ^	F	p	Δ
Anet^[400–400]^	16.96	15.24	9.50	< 0.001**	decrease
${Anet}_{control}^{\left[400–400\right]}$	16.28	16.28	1.01e^-6^	0.999	no change
Anet^[400–800]^	16.96	22.24	63.35	< 0.001**	increase
${Anet}_{control}^{\left[400–800\right]}$	16.28	21.71	11.13	< 0.001**	increase
A_max_	20.99	18.93	6.12	0.014*	decrease
φ	0.05	0.04	27.56	< 0.001**	decrease
J	155.72	111.70	193.52	< 0.001**	decrease
LCP	42.98	30.70	23.97	< 0.001**	decrease
θ	0.51	0.40	12.91	< 0.001**	decrease
V_cmax_	64.29	48.05	90.69	< 0.001**	decrease
TPU	13.52	9.78	204.43	< 0.001**	decrease
Γ	56.03	54.56	2.22	0.14	decrease
R_dark_	2.05	1.35	33.57	< 0.001**	decrease

[400–400] = response in net photosynthesis measured at 400ppm and 400ppm in the cuvette at the first treatment point and second treatment point respectively

[400–800] = response in net photosynthesis measured at 400ppm and 800ppm in the cuvette at the first treatment point and second treatment point respectively

control = trait measured on control plants grown at ambient (aCO_2_) conditions in both treatments (no CO_2_ treatment)

μ = least-square treatment means

F = F-statistic for the linear model

p = p-value for linear model fit of treatment as a fixed effect

Δ = direction of change (slope) between first and second treatment

* = significant at p < 0.05

** = significant at p < 0.001

**Fig 4:**
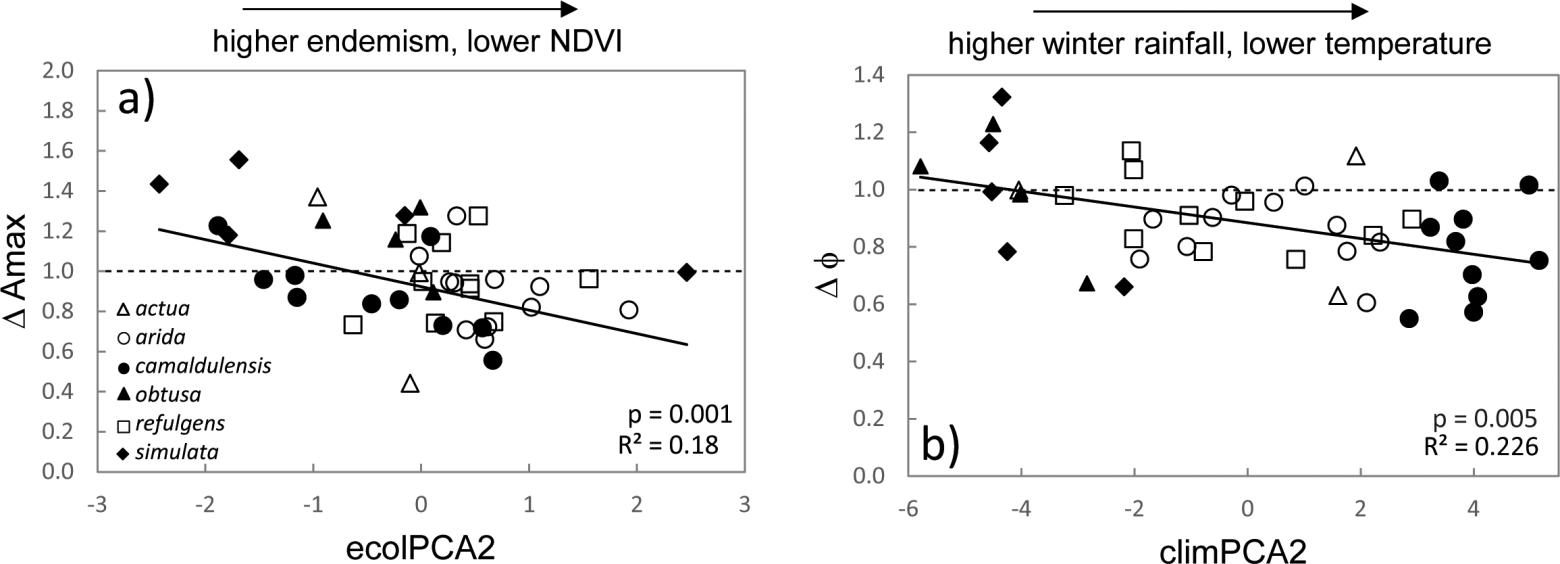
Associations between photosynthetic responses, a) ΔAmax and b) Δφ, between CO_2_ regimes for test plants. The dashed lines at Δtrait = 1 is the expected response ratio if no change is observed between treatment.

**Fig 5:**
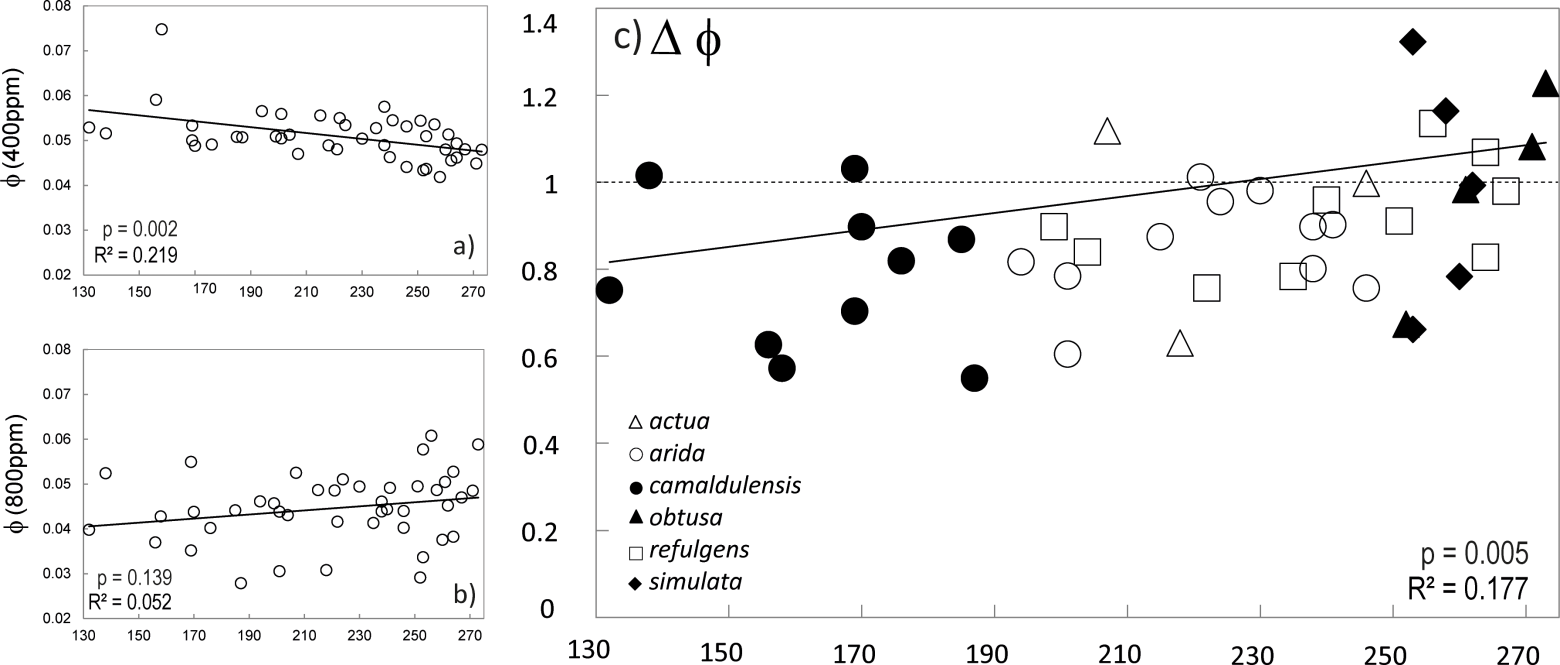
Association between quantum yield (φ) and mean annual temperature at site of origin across for the a) ambient and b) elevated [CO_2_] treatments, and c) relationship between φresponse ratio (Δφ) and provenance mean annual temperature. Units for WorldClim temperature data are in ^o^C*10.

Genotype by environment (G × E) interaction was suggested from intersection or scale change of provenance level reaction norms (Δ trait). Departure of cross treatment genetic correlation (gCor) from unity supported a G × E interaction in response to CO_2_ treatment for quantum yield (φ) where the correlation estimate departed from one within one standard error (Table 5). For all other traits the model converged but the estimate was at the boundary of the parameter space and standard errors could not be estimated. Gene by environment interaction was also suggested for a further five traits based on lack of significant spearman’s rank correlation for provenance BLUEs across treatments. Association of provenance level trait reaction norms and environment at provenance site of origin for traits where G × E was implicated suggest adaptive evolution (or adaptive plasticity) as a possible driver of variation in response across CO_2_ treatments for (Figs 4 and 5; Table 6). The strongest evidence for adaptive plasticity was detected based on environmental association with quantum yield (φ). In this case a downward shift in φunder elevated CO_2_ was detected in provenances originating from cooler climates, with lower summer rainfall and irradiance, relative to populations from northern latitudes (Figs 4B and 5). Response in light saturated net photosynthesis (A_max_) was also negatively associated with ecolPC2, indicating provenances with higher primary productivity (based on higher NDVI) tended to respond more positively to CO_2_ increase relative to other sites (Fig 4A).

**Table 5:** Tests for provenance by CO_2_ interaction (G×E).

trait	gCor	r_s_	r_p_	G × E
Anet^[400–400]^	^˗^	0.04	0.69**	suggested
A_max_	^˗^	0.22	0.16**	suggested
φ	0.101±0.76*	0.02	0.92**	supported
J	^˗^	0.44	0.004	no interaction
LCP	^˗^	0.11	0.49**	suggested
θ	^˗^	0.04	0.83**	suggested
V_cmax_	-	0.22	0.15**	suggested
TPU	^˗^	0.37	0.02	no interaction
Γ	^˗^	0.28	0.07	no interaction
R_dark_	^˗^	0.22	0.15**	no interaction

[400–400] = response in net photosynthesis measured at 400ppm in the cuvette in both treatments

gCor = cross treatment genetic correlation estimated from variance components while fitting provenance as a random effect within each treatment ± standard error

* = correlation significantly departed from unity within one standard error (GxE supported)

r_s_ = Spearman coefficient of rank correlation for provenance BLUEs

r_p_ = p-value for significant Spearman rank correlation

** = rank correlation not significant (GxE suggested)

˗ = correlation estimate at boundary of the parameter space and standard error could not be estimated

**Table 6:** Association of trait response, where G×E was supported or suggested, and multivariate environmental parameters at provenance site of origin.

Δtrait	environment	slope	R^2^	F	p	F^†^	p^†^
Anet^[400–400]^	ecolPC2	-0.111	0.14	6.70	0.013*	6.40	0.016*
A_max_	ecolPC2	-0.117	0.18	8.99	0.005**	11.07	0.002**
φ	climPC2	-0.027	0.23	11.99	0.001***	12.43	0.002**
LCP	ecolPC2	-0.146	0.12	5.54	0.023*	4.93	0.032*
θ	geolPC2	0.153	0.11	5.28	0.027*	5.36	0.026*

[400–400] = response in net photosynthesis measured at 400ppm in the cuvette at both time points

R^2^ = square of Pearson’s R for the model

F = ANOVA F-statistic

p = probability of model

**†** estimate based on a linear model controlling for geographic coordinates

* = significant at p < 0.05

** significant at p < 0.01

*** significant at p < 0.001

## Discussion

### Photosynthetic variation at ambient CO_2_

Adaptive clines along environmental gradients for growth and phenology traits are widely observed in trees [49,50,51]. Although genetic variation in photosynthetic traits has been reported in crops and undomesticated plants [52], natural variation among populations has less commonly been examined in trees, or indeed eucalypts [22,53], and consequently our understanding of the extent of local adaptation in these traits in widespread tree species is limited. Here we shed light on genetic factors contributing to variation in photosynthetic traits based on significant subspecies variation for several biochemical drivers of photosynthesis. In several cases associations with environment at site of origin suggest clines in trait variation could have resulted from local adaptation.

Associations with environment for quantum yield (φ) and other traits (A_max_, TPU and J) suggest increased photosynthetic capacity under well-watered conditions in seedlings originating from cooler, temperate climates with decreased irradiance, possibly reflecting an adaptive cline (Fig 2A). Adaptive clines in some of these photosynthesis traits have been suggested in other organisms, for example increased drought tolerance has been affiliated with decreased φ in European beech (Aranda et al. 2014). Light saturated photosynthesis (A_max_) has been shown to be highest in poplar and spruce originating from cooler habitats [54,55], while temperature sensitivity in trees and other photosynthetic organisms suggest φ can be tightly optimised to suit local conditions [56,57,58,59]. Quantifiable adaptation of phenotype to local environment could have implications for forests under forecasted climate redistributions. For example, persistence of locally adapted populations could be impacted if phenotypes linked with productivity, such as photosynthesis, are maladapted under future conditions [16,60]. It has therefore been recommended that patterns of phenotypic and genetic adaptation should be applied to improve prediction of forest responses to climate change [61]. While it is unclear whether our findings in seedlings would extrapolate to mature forests [48,62], or indeed whether the observed variation in photosynthetic traits would directly link to variation in productivity or fitness [63,64,65], it does suggest a basis for further consideration of photosynthetic traits when assessing adaptive potential.

### CO_2_ response and adaptive plasticity

Overall response measured at 400ppm in both ambient and elevated treatments indicated down regulation of net photosynthesis (A_net_) at elevated CO_2_, but not in control plants (Table 4). The same trend was detected when assessing overall A_net_ (at 400ppm) taken from the *A-C*_*i*_ curve. Down regulation of net photosynthesis also coincided with a decrease in biochemical processes of photosynthesis, pointing to a general down turn in photosynthetic activity under elevated CO_2_. This is consistent with accounts of acclimation of photosynthesis to growth under elevated CO_2_ in other plants [66,67,68,69,70], which has been suggested to result from a combination of nutrient and sink limitations [71].

Evidence for a G×CO_2_ interaction was detected for a single biochemical parameter of photosynthesis, quantum yield (φ), but was suggested for other photosynthetic traits. Previously, the effect of genotype on photosynthesis in response to CO_2_ was found to be limited in a less diverse sampling within the sub species *E*. *c*. *camaldulensis* [22], though genetic variation in plasticity of leaf physiological traits has been detected in other eucalypts [38,72,73,74]. Correlations detected between Δφ and climate factors loading to climPC2 indicate that adaptation to local environment could be a determinant of responsiveness of quantum yield under CO_2_ enrichment (Table 6). The cline in Δφ implied downregulation of photon conversion during photosynthesis under elevated CO_2_ in provenances originating from temperate (*sub sp*. *camaldulensis*) or arid zones (*sub sp*. *arida* and *refulgens*) which on average have cooler climates, with lower summer rainfall and irradiance, relative to provenances at more northern latitudes (*sub sp*. *obtusa*, *simulata* and *refulgens*) (Fig 4B). Component loadings identified mean annual temperature as the variable most strongly contributing to this cline (Table 5C, S3 Table). The cline in responsiveness along this temperature gradient was inversely correlated with provenance level trends in φ detected under ambient conditions (Figs 3 and 5A), suggesting that CO_2_ responsiveness of φ may be constrained by pre-existing environmentally prescribed genetic adaptation of this trait to temperature (and other factors) *in situ*. We note that shading or other factors related to growth rate are not implicated because φin the elevated treatment showed no correlation with final plant height (R^2^ = 0.004, p = 0.68). Genetic adaptation of plasticity (or adaptive plasticity) in quantum yield (φ) has been proposed in other plants, though this is the first report relating φ to variation in CO_2_ response [75,76]. The biological mechanism by which temperature optimisation of φ could impact this trait under higher CO_2_ levels is not resolved here, but warrants further investigation.

Our findings point to the potential for genetic variation among populations, possibly in response to environmental adaptation, to constrain responsiveness to CO_2_ enrichment in at least one photosynthetic trait in *E*. *camaldulensis* seedlings. This is a potentially significant finding as it suggests that photosynthetic efficiency of populations could respond differently to CO_2_ enrichment and in ways not predicted from generalised species responses. Although photosynthetic variation has been directly linked to fitness in some plants [77,78], the implications of adaptive plasticity in CO_2_ response for productivity of mature forests or plantations under prolonged CO_2_ enrichment remain unclear [63,64,65]. A more exhaustive assessment linking variation in photosynthesis response to CO_2_ with growth and fitness traits, including trees at later developmental stages and over longer exposure periods, is needed to better understand this. In addition, interactions between CO_2_ response and other climate variables were not assessed here but will need to be considered [79,80,81]. Future experiments addressing G×CO_2_ would benefit from increased replication of genotypes within provenances to improve power to detect genetic effects for highly variable instantaneous, and to a lesser extent integrated, estimates of photosynthesis.

## Supporting information

S1 Fig Subspecies biplot of the first two discriminant functions determined PCA of individual trait values under ambient [CO_2_]. Discriminant function coefficients are plotted for each PC, scaled to the discriminant function axes, indicating their relative importance in defining subspecies groups.
(TIF)

S2 Fig Representative A-Ci (a-b) and light (c-d) curves for a subset of eight genotypes spanning the range of the φ parameter estimate in the ambient and elevated CO_2_ treatments indicate the quality of data from which biochemical parameters of photosynthesis were estimated. φ estimates for individual trees are as follows: Balranald—tree 1 (0.033), Palmer River—tree 7 (0.044), Nyngan—tree 7 (0.047), Arrowsmith Lake—tree 1 (0.050); Minderoo–tree 2 (0.053), Station Creek–tree 1 (0.055), Nullagine Creek–tree 2 (0.058), Station Creek–tree 3 (0.067). Inset, initial slope between 0 and 500 ppm [CO_2_] for the A-Ci curve, and 0 and 500 photon flux for the light curve.
(TIF)

S3 Fig Provenance level trait-trait correlation plots for photosynthetic traits, with the following naming convention changes: f = quantum yield (φ), q = curvature of the light-response curve (θ) and G = compensation point (Γ). (TIFF)

S4 Fig Box plots illustrating the distribution of individual plant response ratios for A_net_ (elevated A_net_^[400]^/ambient A_net_^[400]^), for all control and test plants in this study. The distribution of response ratio across samples was not significantly different to 1 (theoretical mean for a distribution based on plants with no CO_2_ response, dashed line), whereas mean response ratio for test plants was significantly greater than 1. Plots present the mean, 1^st^ and 3^rd^ quartiles of the distribution and outliers within whiskers spanning 1.5 times the interquartile range (IQR).
(TIFF)

S1 File Raw trait data for individual genotypes collected across two CO_2_ treatments. (CSV)

S1 Table Photosynthetic parameters measured. (DOCX)

S2 Table Principal component loadings for the first 3 principal components derived from individual plant measurements measured at ambient CO_2_ (400ppm). (DOCX)

S3 Table Principal component loadings for environmental parameters. (DOCX)

S4 Table Principal component loadings for the first 3 principal components derived from trait provenance BLUEs estimated at ambient CO_2_ (400ppm). (DOCX)

S5 Table Provenance level correlations between photosynthetic traits (BLUEs) at ambient CO_2_. (DOCX)
