# Supplementary figures and images for: Photosynthetic variation and responsiveness to CO2 in a widespread riparian tree
Source: PLoS One. 2018 Jan 2;13(1):e0189635. doi: 10.1371/journal.pone.0189635 (PMC5749701; doi:10.1371/journal.pone.0189635)

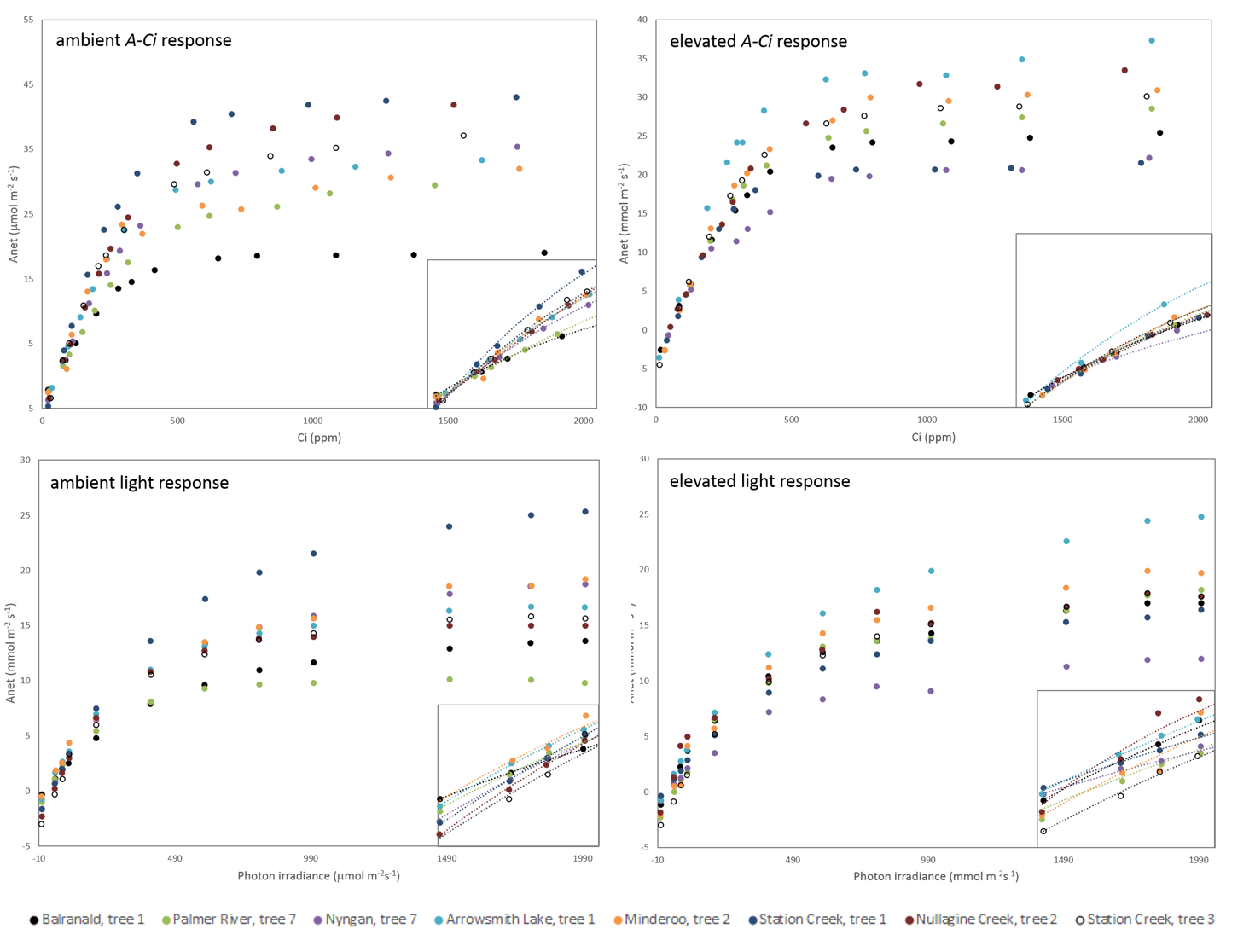

Supplement: S1 Fig — Discriminant function coefficients are plotted for each PC, scaled to the discriminant function axes, indicating their relative importance in defining subspecies groups. (TIF) [file pone.0189635.s001.tif]

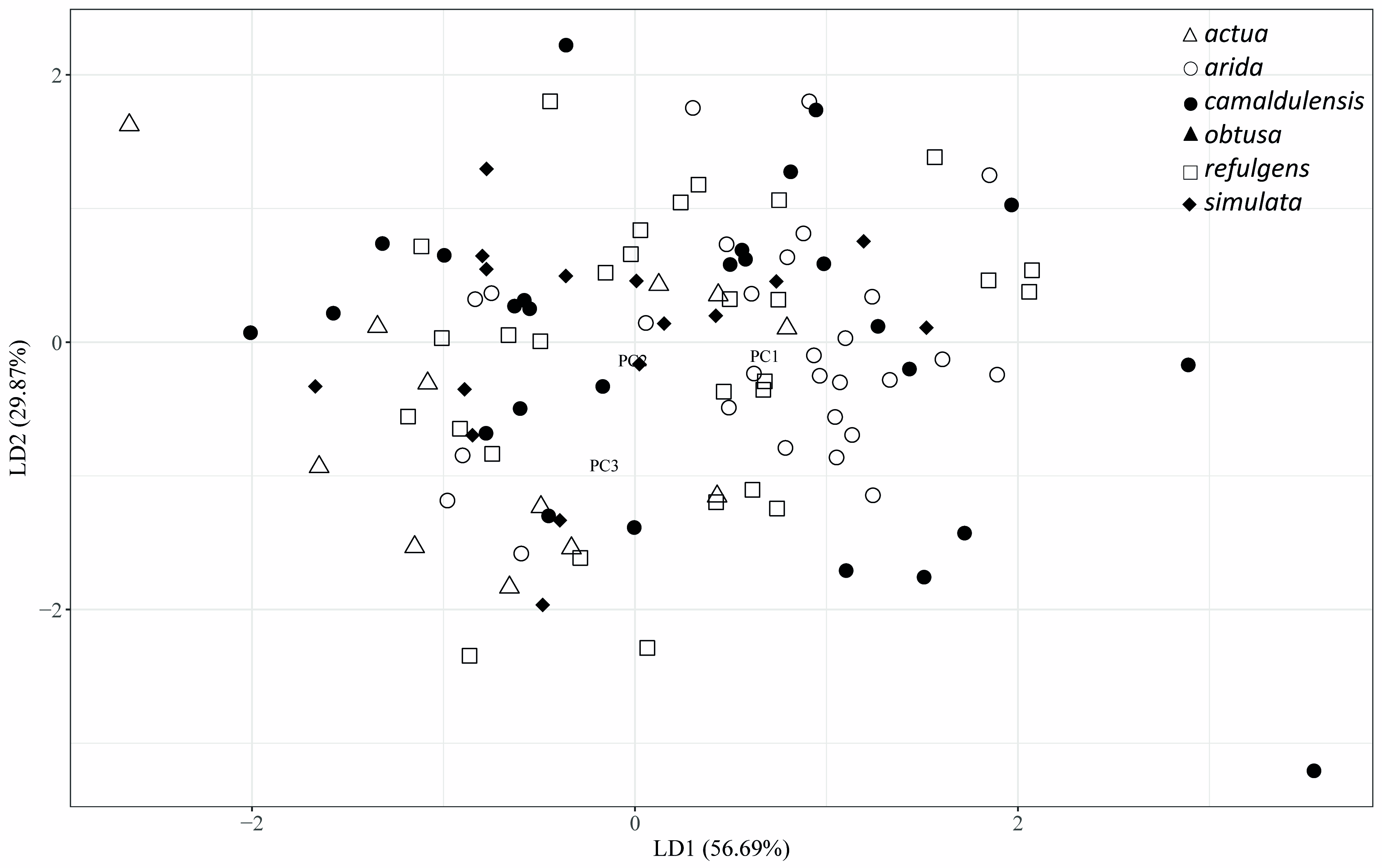

Supplement: S2 Fig — Representative A-Ci (a-b) and light (c-d) curves for a subset of eight genotypes spanning the range of the φ parameter estimate in the ambient and elevated CO2 treatments indicate the quality of data from which biochemical parameters of photosynthesis were estimated. φ estimates for individual trees are as follows: Balranald—tree 1 (0.033), Palmer River—tree 7 (0.044), Nyngan—tree 7 (0.047), Arrowsmith Lake—tree 1 (0.050); Minderoo–tree 2 (0.053), Station Creek–tree 1 (0.055), Nullagine Creek–tree 2 (0.058), Station Creek–tree 3 (0.067). Inset, initial slope between 0 and 500 ppm [CO2] for the A-Ci curve, and 0 and 500 photon flux for the light curve. (TIF) [file pone.0189635.s002.tif]

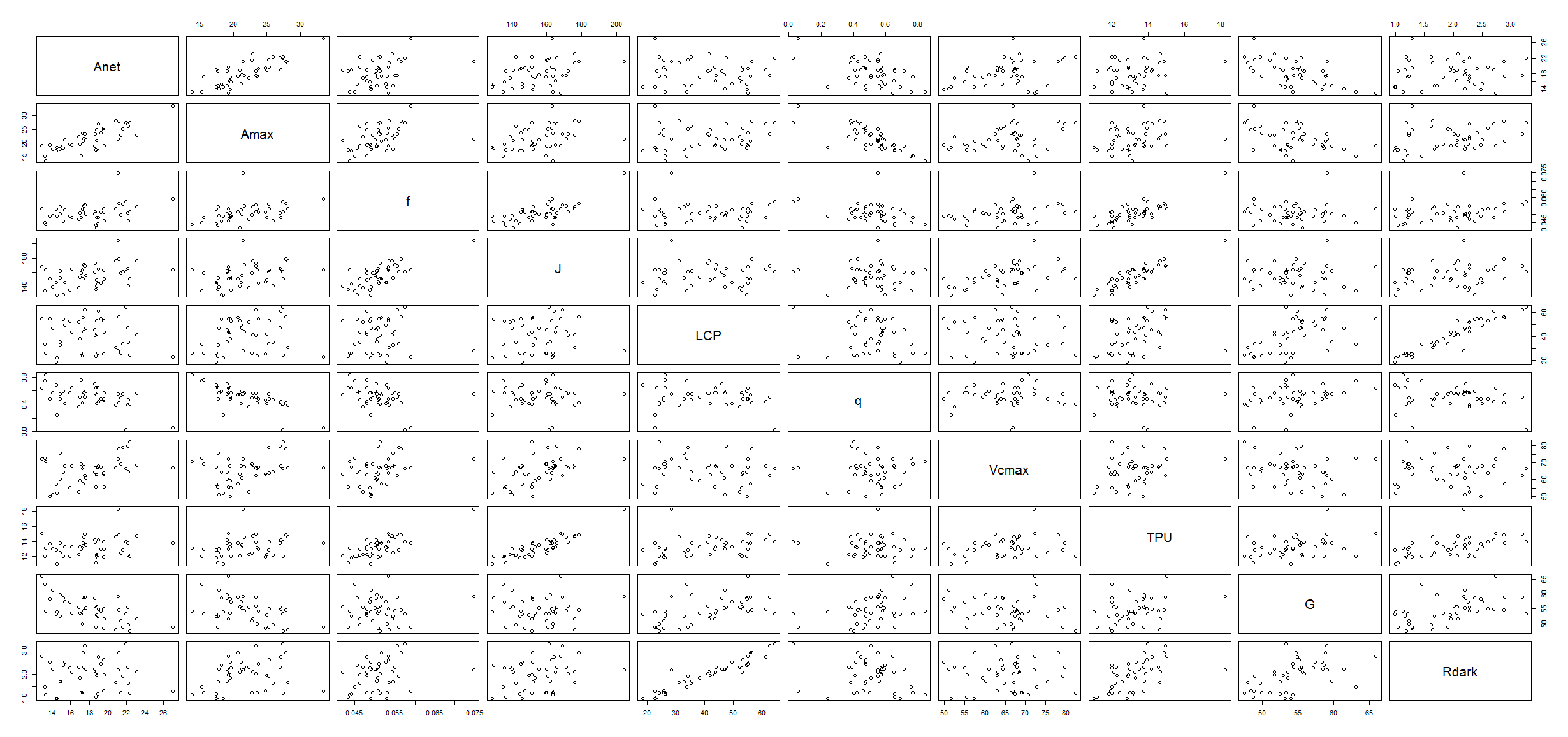

Supplement: S3 Fig — (TIFF) [file pone.0189635.s003.tiff]

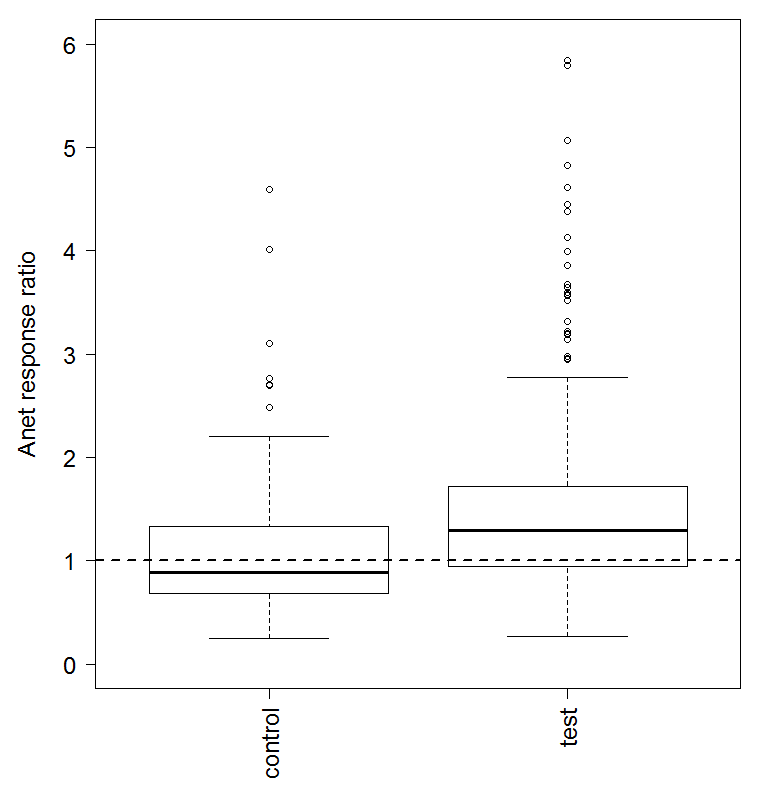

Supplement: S4 Fig — The distribution of response ratio across samples was not significantly different to 1 (theoretical mean for a distribution based on plants with no CO2 response, dashed line), whereas mean response ratio for test plants was significantly greater than 1. Plots present the mean, 1st and 3rd quartiles of the distribution and outliers within whiskers spanning 1.5 times the interquartile range (IQR). (TIFF) [file pone.0189635.s004.tiff]
