# Supplementary material for: Photosynthetic variation and responsiveness to CO2 in a widespread riparian tree
Source: PLoS One. 2018 Jan 2;13(1):e0189635. doi: 10.1371/journal.pone.0189635 (PMC5749701; doi:10.1371/journal.pone.0189635)
Supplement: S1 Table — (DOCX) [file pone.0189635.s006.docx]

| **type** | **curve** | **trait abr.** | **trait** | **further description** |
| --- | --- | --- | --- | --- |
| instantaneous | - | A_net_ | instantaneous light-saturated CO_2_ assimilation rate | - |
| integrated | *A-light* | A_max_ | maximum rate of CO_2_ assimilation | maximum rate of CO_2_ assimilation under light saturation |
| integrated | *A-light* | φ | quantum yield | efficiency of conversion of photons to molecular CO_2_ (photosynthetic efficiency) |
| integrated | *A-C_i_* | J | electron transport rate | maximum rate of carboxylation limited by electron transport |
| integrated | *A-light* | LCP | light compensation point | light intensity where the rate of photosynthesis matches the rate of respiration |
| integrated | *A-light* | θ | curvature of the light-response curve | gradient of photosynthetic capacity |
| integrated | *A-C_i_* | V_cmax_ | carboxylation rate | maximum rate of Rubisco carboxylation |
| integrated | *A-C_i_* | TPU | triose phosphate utilisation | rate of use of triose phosphates |
| integrated | *A-C_i_* | Γ | photorespiratory compensation point | [CO_2_] at which oxygenation proceeds at twice the rate of carboxylation |
| integrated | *A-light* | R_dark_ | dark respiration rate | respiratory CO2 release other than by photorespiration |
