# Supplementary material for: Photosynthetic variation and responsiveness to CO2 in a widespread riparian tree
Source: PLoS One. 2018 Jan 2;13(1):e0189635. doi: 10.1371/journal.pone.0189635 (PMC5749701; doi:10.1371/journal.pone.0189635)
Supplement: S2 Table — (DOCX) [file pone.0189635.s007.docx]

| **trait** | **PC1** | **PC2** | **PC3** |
| --- | --- | --- | --- |
| A_net_ | 0.144 | -0.305 | 0.161 |
| A_max_ | 0.661 | -0.451 | -0.489 |
| φ | 0.721 | -0.201 | -0.130 |
| J | 0.781 | -0.246 | 0.483 |
| LCP | 0.505 | 0.756 | -0.214 |
| θ | -0.376 | 0.297 | 0.823 |
| V_cmax_ | 0.537 | -0.314 | 0.405 |
| TPU | 0.831 | 0.038 | 0.417 |
| Γ | 0.125 | 0.833 | 0.102 |
| R_dark_ | 0.679 | 0.652 | -0.208 |
| % | 34.400 | 57.158 | 73.461 |
