# Supplementary material for: Photosynthetic variation and responsiveness to CO2 in a widespread riparian tree
Source: PLoS One. 2018 Jan 2;13(1):e0189635. doi: 10.1371/journal.pone.0189635 (PMC5749701; doi:10.1371/journal.pone.0189635)
Supplement: S3 Table — (DOCX) [file pone.0189635.s008.docx]

|  |  |  | |
| --- | --- | --- | --- |
| **type** | **environment** | **PC 1** | **PC 2** |
| climate | Distance - to coast | -0.564 | 0.273 |
|  | Evaporation - annual mean | -0.879 | -0.344 |
|  | Evaporation - month max | -0.906 | 0.13 |
|  | Evaporation - month min | -0.325 | -0.9 |
|  | Evaporation - variability | -0.866 | -0.052 |
|  | Humidity - annual mean relative | 0.907 | 0.269 |
|  | Vapour pressure deficit - annual mean | -0.757 | -0.523 |
|  | Precipitation deficit - annual mean | 0.935 | 0.012 |
|  | Precipitation deficit - month max | 0.891 | -0.075 |
|  | Precipitation deficit - month min | 0.887 | -0.189 |
|  | Runoff - average | 0.38 | -0.744 |
|  | WorldClim: Precipitation - annual | 0.765 | -0.446 |
|  | WorldClim: Precipitation - coldest quarter | 0.384 | 0.705 |
|  | WorldClim: Precipitation - seasonality | 0.099 | -0.921 |
|  | WorldClim: Precipitation - warmest quarter | 0.523 | -0.634 |
|  | Aridity index - annual mean | 0.964 | -0.011 |
|  | Moisture Index - annual mean | 0.949 | -0.047 |
|  | Moisture Index - coldest quarter mean | 0.542 | 0.708 |
|  | Moisture Index - seasonality | 0.112 | -0.578 |
|  | Moisture Index - warmest quarter mean | 0.579 | -0.662 |
|  | Water deficit - month max | -0.754 | 0.05 |
|  | Water deficit - month mean | -0.81 | 0.235 |
|  | Water deficit - month min | -0.716 | 0.137 |
|  | Water potential - annual mean | 0.851 | -0.186 |
|  | Water potential - month max | 0.887 | -0.194 |
|  | Water stress index - annual mean | 0.972 | 0.024 |
|  | Water stress index - month max | 0.935 | -0.155 |
|  | Water stress index - month min | 0.39 | 0.708 |
|  | Radiation - annual mean | -0.756 | -0.57 |
|  | Temperature - annual max mean | -0.366 | -0.9 |
|  | WorldClim: Temperature - annual mean | -0.259 | -0.944 |
|  | WorldClim: Temperature - driest quarter mean | 0.093 | -0.49 |
|  | WorldClim: Temperature - seasonality | -0.738 | 0.608 |
|  | WorldClim: Temperature - wettest quarter mean | -0.485 | -0.678 |
|  | Wind run - annual mean | -0.145 | 0.506 |
|  | Wind run - month max | -0.047 | 0.455 |
|  | Wind run - month min | -0.279 | 0.479 |
|  | % | 46.30 | 26.07 |
| ecology | NPP Mean | 0.584 | -0.250 |
|  | NDVI Mean | 0.442 | -0.682 |
|  | Endemism | 0.446 | 0.584 |
|  | Species Richness | 0.514 | 0.363 |
|  | % | 62.25 | 24.35 |

**S3 Table** continued

| **environment** | **Variable** | **PC 1** | **PC 2** |
| --- | --- | --- | --- |
| geology | Elevation | 0.111 | -0.266 |
|  | Topographic Slope (degrees) | -0.884 | -0.180 |
|  | Phosphorus - plant-available pre-European | -0.944 | -0.131 |
|  | Carbon store pre-European | -0.922 | -0.105 |
|  | Nitrogen - plant-available pre-European | -0.920 | -0.095 |
|  | Aspect | -0.245 | -0.080 |
|  | Weathering Intensity | 0.453 | 0.101 |
|  | Soil depth | -0.420 | 0.297 |
|  | Erodibility | 0.311 | 0.750 |
|  | Nutrient status | -0.377 | 0.780 |
|  | Soil pedality | -0.375 | 0.848 |
|  | % | 38.21 | 57.61 |
