# Supplementary material for: Photosynthetic variation and responsiveness to CO2 in a widespread riparian tree
Source: PLoS One. 2018 Jan 2;13(1):e0189635. doi: 10.1371/journal.pone.0189635 (PMC5749701; doi:10.1371/journal.pone.0189635)
Supplement: S4 Table — (DOCX) [file pone.0189635.s009.docx]

|  |  | | |
| --- | --- | --- | --- |
| **trait** | **PC 1** | **PC 2** | **PC 3** |
| A_net_ | 0.681 | -0.018 | -0.504 |
| Γ | 0.337 | -0.009 | 0.836 |
| φ | 0.073 | 0.832 | -0.029 |
| A_max_ | 0.042 | 0.652 | -0.470 |
| θ | 0.132 | -0.458 | 0.238 |
| TPU | 0.321 | 0.810 | 0.221 |
| V_cmax_ | 0.038 | 0.460 | -0.380 |
| J | 0.199 | 0.816 | -0.113 |
| R_dark_ | 0.611 | 0.440 | 0.512 |
| LCP | 0.580 | 0.213 | 0.548 |
| % | 26.67 | 24.87 | 15.90 |

% = percent variance explained by principal components
