# Supplementary material for: Photosynthetic variation and responsiveness to CO2 in a widespread riparian tree
Source: PLoS One. 2018 Jan 2;13(1):e0189635. doi: 10.1371/journal.pone.0189635 (PMC5749701; doi:10.1371/journal.pone.0189635)
Supplement: S5 Table — (DOCX) [file pone.0189635.s010.docx]

| **trait** | **trait** | **slope** | **R^2^** | **F** | **p** |
| --- | --- | --- | --- | --- | --- |
| Amax | Anet | 1.05 | 0.65 | 74.78 | 8.71e-11** |
|  | J | 0.1 | 0.13 | 5.94 | 0.019* |
|  | θ | -19.55 | 0.57 | 54.06 | 5.26e-09** |
|  | Vcmax | 0.17 | 0.11 | 4.87 | 0.033* |
| φ | Amax | 5.44e-04 | 0.17 | 8.25 | 0.006** |
|  | Anet | 6.26e-04 | 0.13 | 6.15 | 0.017* |
|  | J | 2.65e-04 | 0.53 | 45.35 | 3.91e-08** |
|  | θ | -0.01 | 0.12 | 5.76 | 0.021* |
|  | TPU | 3.30e-03 | 0.61 | 62.96 | 8.16e-10** |
|  | Vcmax | 2.23e-04 | 0.1 | 4.57 | 0.039* |
| Γ | Amax | -0.45 | 0.2 | 10.16 | 0.002** |
|  | Anet | -0.81 | 0.38 | 25.09 | 1.09e-05** |
|  | LCP | 0.17 | 0.28 | 15.7 | 0.0003*** |
|  | θ | 8.61 | 0.11 | 4.95 | 0.031* |
|  | Rdark | 3.52 | 0.27 | 15.21 | 0.0004*** |
|  | TPU | 1.04 | 0.1 | 4.7 | 0.036* |
| J | Anet | 1.8 | 0.14 | 6.89 | 0.012* |
| θ | Anet | -0.03 | 0.26 | 14.76 | 0.0004*** |
| Rdark | LCP | 0.04 | 0.9 | 362.48 | 5.73e-22** |
| TPU | J | 0.08 | 0.8 | 160.86 | 8.87e-16** |
|  | Rdark | 1.08 | 0.27 | 14.9 | 0.0004*** |
| Vcmax | Anet | 0.94 | 0.14 | 6.92 | 0.012* |
|  | J | 0.25 | 0.23 | 12.27 | 0.001*** |

R^2^ = square of Pearson’s R for the model.

F = ANOVA F-statistic

p = probability of model

* = significant at p < 0.05

** = significant at p < 0.01

** *= significant at p < 0.001

**·** = marginal significance

Anet taken from A_ci_ curve
